# ﻿Two new species of *Fulvifomes* (Hymenochaetales, Basidiomycota) from South America

**DOI:** 10.3897/mycokeys.119.158957

**Published:** 2025-07-08

**Authors:** Jian Chen, Yuan Yuan, Kai-Yue Luo, Josef Vlasák

**Affiliations:** 1 State Key Laboratory of Efficient Production of Forest Resources, School of Ecology and Nature Conservation, Beijing Forestry University, Beijing 100083, China Beijing Forestry University Beijing China; 2 Biology Centre of the Academy of Sciences of the Czech Republic, České Budějovice, Czech Republic Biology Centre of the Academy of Sciences of the Czech Republic České Budějovice Czech Republic

**Keywords:** Hymenochaetales, phylogenetic analysis, taxonomy, wood-decaying fungi

## Abstract

Two new species of *Fulvifomes* are described from specimens collected in South America (Ecuador and Bolivia) based on morphological and molecular evidence. The species *Fulvifomesbolivianus* is characterized by perennial, pileate, solitary basidiocarp; ungulate pilei; olivaceous, encrusted, rough, concentrically sulcate, irregularly cracked pileal surface; umber and glossy pore surface; circular pores measuring 7–8 per mm; thick and entire dissepiments; a dimitic hyphal system; and subglobose basidiospores measuring 4.7–5.4 × 4–4.5 µm. *Fulvifomesfragilis* is characterized by annual, pileate, imbricate basidiocarps; curry yellow to yellowish brown pileal surface; yellowish brown and narrow pileal margin; yellow and rugged pore surface; circular to elliptical pores measuring 5–6 per mm; thick and entire dissepiments; a dimitic hyphal system; and broadly ellipsoid basidiospores measuring 3.9–5.1 × 3–3.7 µm. Phylogenetically, the two new species formed two distinct lineages in the *Fulvifomes* clade based on internal transcribed spacer (ITS) and nuclear-encoded large subunit rRNA gene (nLSU) sequences. The differences between the new species and morphologically similar and phylogenetically related species are also discussed.

## ﻿Introduction

Hymenochaetaceae is an important family of wood-inhabiting fungi. Species diversity and taxonomy of the family have been systematically studied, and around 920 species have been recognized in the family (Dai 2010; Wu et al. 2022; Dong et al. 2024; Liu et al. 2025). Some very important medicinal fungi belong to this family (Chu et al. 2023; Lv et al. 2023; Yang et al. 2023; Lang et al. 2024; Ma et al. 2024; Zhang et al. 2024). *Fulvifomes* Murrill is a genus belonging to Hymenochaetaceae, and it was described based on *F.robiniae* (Murrill) Murrill, grouping species of polypores with perennial basidiocarps, brown and woody context, and colored basidiospores (Murrill 1903; Murrill 1914). The genus was treated as a synonym of *Phellinus* Quél. for several decades (Gilbertson and Ryvarden 1986; Larsen and Cobb-Poulle 1990) until Wagner and Fischer (2002) confirmed *Fulvifomes* as an independent genus based on nuclear-encoded large subunit rRNA gene (nLSU) sequences. Due to uniform morphology, traditional *Fulvifomes* classification only enables definition of a limited number of species based on a few distinct features (Dai 2010; Hattori et al. 2014; Salvador-Montoya et al. 2018; Wu et al. 2022). Based on molecular and morphological analyses, Zhou (2014) revised the definition of *Fulvifomes*. The genus is characterized by basidiomata that are annual to perennial, pileate or substipitate, effused-reflexed or substipitate with a contracted base, solitary or imbricate, and corky to woody hard; pileal surface tomentose or glabrous, with or without a crust; context homogenous or duplex; hyphal system monomitic or dimitic; generative hyphae simple septate; setal elements absent; basidiospores subglobose to ellipsoid, yellowish to brown, fairly thick- to thick-walled, smooth; causing a white rot (Zhou 2014; Zhou 2015; Wu et al. 2022). Most species of *Fulvifomes* grow on dead angiosperm trees or wood, although a few can inhabit living trees (Ji et al. 2017; Wu et al. 2022). Most species of the genus have been found in tropical or subtropical areas of Asia, America, and Africa (Ediriweera et al. 2014; Olou et al. 2019; Fatima et al. 2023; Zhou et al. 2023; Zhao et al. 2024).

Moreover, many species of *Fulvifomes* are forest pathogens, and some species have therapeutic properties (Dai et al. 2007; Wu et al. 2019; Yuan et al. 2023). Three previously undescribed limonoids (fulvifomins A–C) were isolated from *Fulvifomesxylocarpicola* T. Hatt., Sakay. & E.B.G. Jones, of which fulvifomin B exhibited antimalarial and antitubercular activities (Isaka et al. 2021). To date, more than 50 species have been recorded in *Fulvifomes* (Wu et al. 2022), but there have been only a few reports on the isolation of bioactive compounds from *Fulvifomes* species (Ding et al. 2019; Isaka et al. 2021; Lin et al. 2024). One contributing factor may be the relatively recent recognition of *Fulvifomes* as an independent genus. Presently, *Fulvifomes* is widely accepted as an independent genus in Hymenochaetaceae, and several new taxa of the genus have been detected based on molecular and morphological evidence (Martínez et al. 2023; Zhou et al. 2023; Suh et al. 2024).

During an investigation on the wood-inhabiting fungi from South America (Ecuador and Bolivia), samples were collected with morphological characteristics fitting *Fulvifomes*. Phylogenetically, they formed independent lineages within *Fulvifomes*. Therefore, we describe two new species of *Fulvifomes* in the present paper.

## ﻿Materials and methods

### ﻿Morphological studies

The studied specimens are deposited in the Fungarium of the Institute of Microbiology, Beijing Forestry University (BJFC), Beijing, China. Morphological descriptions were based on field notes and voucher specimens. The microscopic analysis follows Wu et al. (2022), and the special color terms follow Anonymous (1969) and Petersen (1996). Microscopic features, measurements, and illustrations were made from slide preparations stained with Melzer’s reagent (IKI) and Cotton Blue (CB). The sections were studied at magnifications up to ×1,000 using a Nikon Eclipse 80i microscope with phase contrast illumination. The following abbreviations are used: **KOH** = 5% potassium hydroxide, **IKI** = Melzer’s reagent, **IKI[+]** = dextrinoid, **IKI–** = both inamyloid and non-dextrinoid, **CB** = Cotton Blue, **CB+** = cyanophilous, **CB–** = weakly acyanophilous; **L** = arithmetic average of spore length, **W** = arithmetic average of spore width, **Q** = L/W ratios, and **n** = number of basidiospores measured from a given number of specimens. Basidiospores were measured from sections cut from the tubes.

### ﻿DNA extraction, amplification, and sequencing

The CTAB rapid plant genome extraction kit-DN14 (Aidlab Biotechnologies Co., Ltd., Beijing) was used to obtain DNA from dried specimens. The primer pair ITS4 and ITS5 was used for amplification of the ITS region, while the primer pair LR0R and LR7 was used to amplify the nuclear large subunit ribosomal RNA gene (nLSU) (White et al. 1990). The PCR procedure for ITS was as follows: initial denaturation at 95 °C for 3 min, followed by 35 cycles at 94 °C for 40 s, 54 °C for 45 s, and 72 °C for 1 min, and a final extension at 72 °C for 10 min. The PCR procedure for nLSU was as follows: initial denaturation at 94 °C for 1 min, followed by 35 cycles at 94 °C for 30 s, 50 °C for 1 min, and 72 °C for 1.5 min, and a final extension at 72 °C for 10 min. The PCR products were purified and sequenced at the Beijing Genomics Institute, China, with the same primers. Reference ITS and nLSU sequences of *Fulvifomes* were downloaded from GenBank according to Wu et al. (2022) and Suh et al. (2024). All sequences analyzed in this study are listed in Table 1. Both ITS and nLSU datasets were aligned using MAFFT v. 7 (Katoh and Standley 2013) under the G-INS-i option and manually proofread in BioEdit (Hall 1999). Alignments were subsequently spliced and transformed into formats in Mesquite v. 3.2 (Maddison and Maddison 2017).

**Table 1.** Information for the sequences used in this study.

Species name	Sample no.	Location	GenBank accessions no.		References
			ITS	nLSU	
*Flaviporellussplitgerberi*	JV 1908/6	French Guiana	MZ484525	MZ437386	Wu et al. (2022)
*Fomitiporellacaryophylli*	CBS 448.76	India	AY558611	AY059021	Jin et al. (2005)
*Fulvifomesacaciae*	JV 0312/23.4-J	USA	OP828594	OP828596	Zhou et al. (2023)
*Fulvifomesacaciae*	JV 2203/71	Costa Rica	OP828595	OP828596	Zhou et al. (2023)
*Fulvifomesazonatus*	Cui 8452	China	MH390417	MH390396	Wu et al. (2022)
*Fulvifomesazonatus*	Dai 17470	China	MH390418	MH390395	Wu et al. (2022)
***Fulvifomesbolivianus***	**JV 2311/6-J**	**Bolivia**	**PV749018**	—	**Present study**
***Fulvifomesbolivianus***	**JV 2411/15J**	**Bolivia**	**PV749017**	—	**Present study**
*Fulvifomesboninensis*	FFPRI 421009	Japan	LC315786	LC315777	Hattori et al. (2022)
*Fulvifomescaligoporus*	Dai 17668	China	MH390420	MH390390	Wu et al. (2022)
*Fulvifomescaligoporus*	Dai 17660	China	MH390421	MH390391	Wu et al. (2022)
*Fulvifomescentroamericanus*	JV 0611/III	Guatemala	KX960763	KX960764	Ji et al. (2017)
*Fulvifomescentroamericanus*	JV 0611/8P	USA	KX960757	—	Ji et al. (2017)
*Fulvifomescoffeatoporus*	JV0904_1	USA	KX960762	KX960765	Wu et al. (2022)
*Fulvifomescoffeatoporus*	JV0312_24.10J	USA	KX960760	KX960766	Wu et al. (2022)
*Fulvifomescoffeatoporus*	JV1008_21	USA	KX960761	KX960767	Wu et al. (2022)
*Fulvifomescostaricensis*	JV 1407/87	Costa Rica	MH390412	MH390387	Wu et al. (2022)
*Fulvifomescostaricensis*	JV 1408/14	Costa Rica	MH390413	MH390385	Wu et al. (2022)
*Fulvifomescostaricensis*	JV 1607/103	Costa Rica	MH390414	MH390386	Wu et al. (2022)
*Fulvifomesdracaenicola*	Dai 22097	China	MW559800	MW559805	Wu et al. (2022)
*Fulvifomesdracaenicola*	Dai 22093	China	MW559799	MW559804	Wu et al. (2022)
*Fulvifomeselaeodendri*	CMW 47808	South Africa	MH599093	MH599131	Wu et al. (2022)
*Fulvifomeselaeodendri*	CMW 47825	South Africa	MH599094	MH599134	Wu et al. (2022)
*Fulvifomeselaeodendri*	CMW 47909	South Africa	MH599096	MH599132	Wu et al. (2022)
*Fulvifomeselaeodendri*	CMW 48154	South Africa	MH599097	MH599135	Wu et al. (2022)
*Fulvifomeselaeodendri*	CMW 48610	South Africa	MH599095	MH599133	Wu et al. (2022)
*Fulvifomesfastuosus*	LWZ 20140731-13	Thailand	KR905674	KR905668	Zhou (2015)
*Fulvifomesfastuosus*	LWZ 20140718-29	Thailand	KR905673	—	Zhou (2015)
*Fulvifomesfastuosus*	Dai 18292	Vietnam	MH390411	MH390381	Wu et al. (2022)
*Fulvifomesfloridanus*	JV 0904/65	USA	MH390422	—	Wu et al. (2022)
*Fulvifomesfloridanus*	JV 0312/23.1	USA	MH390423	—	Wu et al. (2022)
*Fulvifomesfloridanus*	JV 0904/76	USA	MH390424	MH390388	Wu et al. (2022)
***Fulvifomesfragilis***	**JV 2402/50-J-1**	**Ecuador**	**PV368085**	**PV368092**	**Present study**
***Fulvifomesfragilis***	**JV 2402/50-J-2**	**Ecuador**	**PV368086**	—	**Present study**
*Fulvifomesgrenadensis*	JV 1212/2J	USA	KX960756	—	Ji et al. (2017)
*Fulvifomesgrenadensis*	1607/66	Costa Rica	KX960758	—	Ji et al. (2017)
*Fulvifomeshainanensis*	Dai 11573	China	KC879263	JX866779	Zhou (2014)
*Fulvifomeshalophilus*	XG 4	Thailand	JX104705	JX104752	Wu et al. (2022)
*Fulvifomeshalophilus*	JV 1502/4	USA	MH390427	MH390392	Wu et al. (2022)
*Fulvifomesimazekii*	FFPRI 421006	Japan	LC315787	LC315778	Hattori et al. (2022)
*Fulvifomesimazekii*	FFPRI 421007	Japan	LC315788	LC315779	Hattori et al. (2022)
*Fulvifomesimbricatus*	LWZ 20140728-16	Thailand	KR905677	KR905670	Zhou (2015)
*Fulvifomesimbricatus*	LWZ 20140729-25	Thailand	KR905678	—	Zhou (2015)
*Fulvifomesimbricatus*	LWZ 20140729-26	Thailand	KR905679	KR905671	Zhou (2015)
*Fulvifomesindicus*	Yuan 5932	China	KC879261	JX866777	Zhou (2014)
*Fulvifomesindicus*	O 25034	Zimbabwe	KC879262	KC879259	Wu et al. (2022)
*Fulvifomesjawadhuvensis*	SKM-JMR5	India	MW040079	MW048886	GenBank
*Fulvifomesjouzaii*	JV 1504/16	Costa Rica	MH390425	MH390400	Wu et al. (2022)
*Fulvifomesjouzaii*	JV 1504/39	Costa Rica	MH390426	—	Wu et al. (2022)
*Fulvifomeskawakamii*	PPT152	Brazil	MH048095	MH048085	Zhou (2015)
*Fulvifomeskawakamii*	CBS 428.86	USA	—	AY059028	Wagner and Fischer (2002)
*Fulvifomeslabyrinthus*	SFC20160126-30	FS Micronesia	OR168711	—	Suh et al. (2024)
*Fulvifomeslabyrinthus*	SFC20160126-34	FS Micronesia	OR168712	—	Suh et al. (2024)
*Fulvifomeslloydii*	Dai 10809	China	MH390428	MH390378	Wu et al. (2022)
*Fulvifomeslloydii*	Dai 9642	China	MH390429	MH390379	Wu et al. (2022)
*Fulvifomeslloydii*	Dai 11978	China	MH390430	MH390380	Wu et al. (2022)
*Fulvifomesluteoumbrinus*	CBS 296.56	USA	AY558603	AY059051	Wagner and Fischer (2002)
*Fulvifomesmangroviensis*	KSM-MP1	Tamil Nadu	MW040083	MW048909	Tan et al. (2022)
*Fulvifomesmangroviensis*	KSM-MP12a	Tamil Nadu	OM987221	OM897222	Tan et al. (2022)
*Fulvifomesmerrillii*	6Kout	Thailand	MH390416	MH390383	Wu et al. (2022)
*Fulvifomesmerrillii*	Dai 12094	China	MH390415	MH390376	Wu et al. (2022)
*Fulvifomesnakasoneae*	JV 1109/62	USA	MH390407	MH390376	Wu et al. (2022)
*Fulvifomesnakasoneae*	JV 0904/68	USA	MH390408	MH390373	Wu et al. (2022)
*Fulvifomesnakasoneae*	JV 1109/77	USA	MH390409	MH390374	Wu et al. (2022)
*Fulvifomesnakasoneae*	JV 0312/22.11	USA	MH390410	MH390375	Wu et al. (2022)
*Fulvifomesnilgheriensis*	CBS 209.36	USA	AY558633	AY059023	Wagner and Fischer (2002)
*Fulvifomesnilgheriensis*	URM 3028	Brazil	MH390431	MH390384	Wu et al. (2022)
*Fulvifomesnonggangensis*	GXU1127	China	MT571504	MT571502	Zheng et al. (2021)
*Fulvifomesnonggangensis*	GXU2254	China	MT571503	MT571501	Zheng et al. (2021)
*Fulvifomesrhizophorus*	SFC20170118-26	FS Micronesia	OR168715	OR168723	Suh et al. (2024)
*Fulvifomesrhizophorus*	SFC20170120-06	FS Micronesia	OR168713	OR168724	Suh et al. (2024)
*Fulvifomesrhizophorus*	SFC20170120-09	FS Micronesia	OR168714	OR168725	Suh et al. (2024)
*Fulvifomesrhytiphloeus*	JV 1704/71	Costa Rica	MZ506738	MZ505207	Wu et al. (2022)
*Fulvifomesrhytiphloeus*	JV 1808/76	French Guiana	MZ506739	MZ505208	Wu et al. (2022)
*Fulvifomesrigidus*	Dai 17496	China	MH390432	MH390398	Wu et al. (2022)
*Fulvifomesrigidus*	Dai 17507	China	MH390433	MH390399	Wu et al. (2022)
*Fulvifomesrimosus*	M 2392655	Australia	MH628255	MH628017	Wu et al. (2022)
*Fulvifomesrobiniae*	CBS 211.36	USA	AY558646	AF411825	GenBank
*Fulvifomesrobiniae*	—	—	EF088656	—	Wagner and Fischer (2002)
*Fulvifomessiamensis*	XG 2	Thailand	JX104709	JX104756	Zhou (2014)
*Fulvifomessiamensis*	Dai 18309	Vietnam	MH390434	MH390389	Wu et al. (2022)
*Fulvifomes* sp.	JV 2402/43-J	Ecuador	PV368087	PV368093	Present study
*Fulvifomessquamosus*	CS385	Peru	MF479268	MF479265	Salvador-Montoya et al. (2018)
*Fulvifomessquamosus*	CS444	Peru	MF479269	MF479264	Salvador-Montoya et al. (2018)
*Fulvifomessubindicus*	Dai 17743	China	MH390435	MH390393	Wu et al. (2022)
*Fulvifomessubindicus*	Cui 13908	China	MH390436	MH390394	Wu et al. (2022)
*Fulvifomessubmerrillii*	Dai 17911	China	MH390405	MH390371	Wu et al. (2022)
*Fulvifomessubmerrillii*	Dai 17917	China	MH390406	MH390372	Wu et al. (2022)
*Fulvifomesthailandicus*	LWZ 20140731-1	Thailand	KR905672	KR905665	Zhou (2015)
*Fulvifomesthiruvannamalaiensis*	MUBL 4013	India	NR_185632	NG_228882	GenBank
*Fulvifomesthiruvannamalaiensis*	SKM-JMR11	India	MZ221598	MZ221600	GenBank
*Fulvifomestubogeneratus*	GXU2468	China	MT580805	MT580800	Zheng et al. (2021)
*Fulvifomestubogeneratus*	GXU2478	China	MT580806	MT580801	Zheng et al. (2021)
*Fulvifomeswrightii*	CTES:568251	Argentina	OQ807192	OQ924560	Martínez et al. (2023)
*Fulvifomeswrightii*	CTES:568252	Argentina	OQ807191	OQ924561	Martínez et al. (2023)
*Fulvifomeswrightii*	CTES:568253	Argentina	OQ807194	OQ924558	Martínez et al. (2023)
*Fulvifomesxylocarpicola*	MU 8	Thailand	JX104676	JX104723	Zhou (2014)
*Inocutistamaricis*	CBS 384.72	Turkmenistan	AY558604	MH872221	Vu et al. (2019)
*Inonotushispidus*	S45	Spain	EU282482	EU282484	GenBank
*Phellinusbetulinus*	CBS 122.40	USA	MH856059	MH867554	Wu et al. (2022)
*Phellinuspopulicola*	CBS 638.75	Finland	MH860960	MH872729	Wu et al. (2022)

**Bold** = new taxa, — refers the data unavailability.

### ﻿Phylogenetic analyses

Phylogenetic analyses were performed using maximum likelihood (ML) and Bayesian inference (BI) methods based on ITS + nLSU datasets. Sequence alignments were deposited in TreeBase (http://purl.org/phylo/treebase; submission ID: 32179; reviewer access URL: http://purl.org/phylo/treebase/phylows/study/TB2:S32179?x-access-code=5f27f003f433eeb85630b37affbf34ef&format=html). *Phellinusbetulinus* (Murrill) Parmasto and *P.populicola* Niemelä were used as outgroups in the phylogeny of *Fulvifomes* (Wu et al. 2022). The best-fit evolutionary model was estimated using jModelTest under Akaike Information Criterion (Guindon and Gascuel 2003; Posada 2008). The ML method was performed using RAxML-HPC2 through the CIPRES Science Gateway (Miller et al. 2010), and branch support (BS) was determined by 1000 bootstrap replicates. The BI method was conducted with MrBayes v. 3.2.6 (Ronquist et al. 2012). Four chains were run for 2 million generations (split deviation frequency value was less than 0.01), and trees were sampled every 100 generations. The first 25% of trees were removed, and the remaining ones were used to construct a 50% majority-rule consensus tree and to calculate Bayesian Posterior Probabilities (BPPs). Phylogenetic trees were visualized using FigTree v. 1.4.3. Branches that received bootstrap support for ML (≥ 75% BS) and BPPs (≥ 0.95 BPPs) were considered to be significantly supported.

## ﻿Results

### ﻿Molecular phylogeny

**Figure 1.**
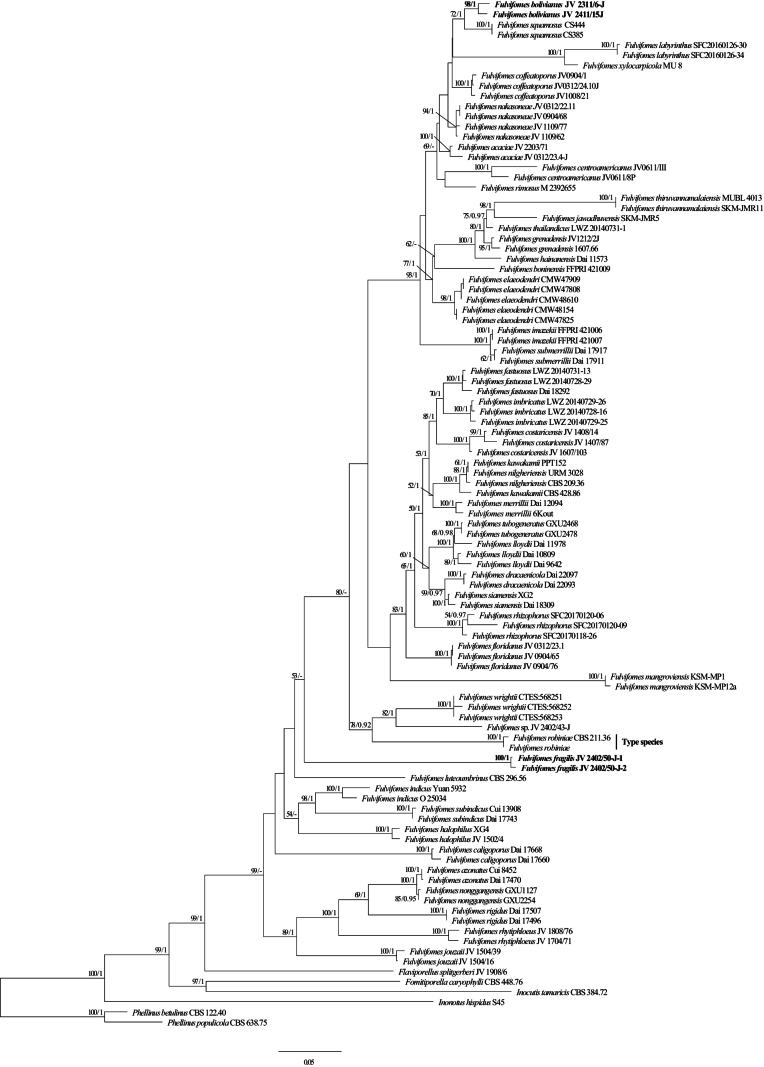
Maximum likelihood tree illustrating the phylogeny of *Fulvifomes* based on the combined dataset of ITS + nLSU sequences. The maximum likelihood bootstrap values (≥50) and Bayesian posterior probability values (≥0.90) are indicated above the branches. The new species is in bold.

In this study, 101 fungal samples representing 51 taxa of *Fulvifomes* were included in the phylogenetic analyses, and two samples of the genus *Phellinus* were used as outgroups. The phylogenetic trees were constructed using a total of 2,061 base pairs (bp) of alignments, including 1,151 bp of ITS and 910 bp of nLSU. The best-fit model applied in the Bayesian analysis was GTR+I+G, lset nst = 6, rates = invgamma, and prset statefreqpr = dirichlet (1, 1, 1, 1). Bayesian analysis resulted in an average standard deviation of split frequencies of 0.009917. The topologies of the generated ML and BI trees are nearly congruent. Thus, only the topologies inferred from the ML method were presented, along with bootstrap values (≥ 50%) from the ML method and BPPs (≥ 0.9) from the BI method. In the phylogeny of *Fulvifomes* (Fig. 1), the two new species each formed an independent lineage with strong support: *Fulvifomesbolivianus* (BS = 98% in ML, BPP = 1.00) and *Fulvifomesfragilis* (BS = 100% in ML, BPP = 1.00).

The ITS sequences showed significant alignments in the NCBI database (https://blast.ncbi.nlm.nih.gov/Blast.cgi) for *Fulvifomesbolivianus*, with all of the top ten sequences having less than 95% similarity, the highest being *F.squamosus* (CS385) at 94.8%. As for *Fulvifomesfragilis*, the similarities of the top ten ITS sequences were less than 90%, and they represent multiple species in *Fulvifomes*. The alignment results in the NCBI database are consistent with our phylogeny (Fig. 1).

## ﻿Taxonomy

### ﻿ Fulvifomes bolivianus

Taxon classificationFungiHymenochaetalesHymenochaetaceae

Jian Chen, Yuan Yuan, K.Y. Luo, Y.C. Dai & Vlasák sp. nov.

47B8821D-5C38-5130-9021-7D6A22D36B66

858480

Figs 2 , 3

#### Diagnosis.

*Fulvifomesbolivianus* is related to *F.squamosus* Salvador-Montoya & Drechsler-Santos, but *F.squamosus* differs from *F.bolivianus* by applanate to triquetrous pilei, a monomitic hyphal system in context, and larger pores (5–6 per mm vs. 7–8 per mm).

**Figure 2.**
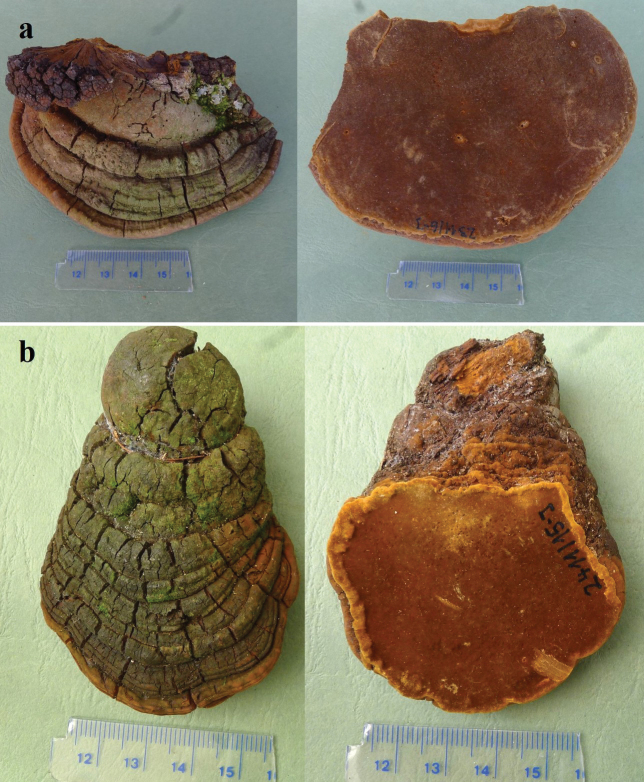
Basidiomata of *Fulvifomesbolivianus* (**a.** Holotype, JV 2311/6-J; **b.** Paratype JV 2411/15J).

#### Holotype.

Bolivia. • St. Cruz Dept., Refugio los Volcanes, altitude 1100 m, dead angiosperm tree, 1.XI.2023, leg J. Vlasák Jr., JV 2311/6-J (BJFC 044745).

**Figure 3.**
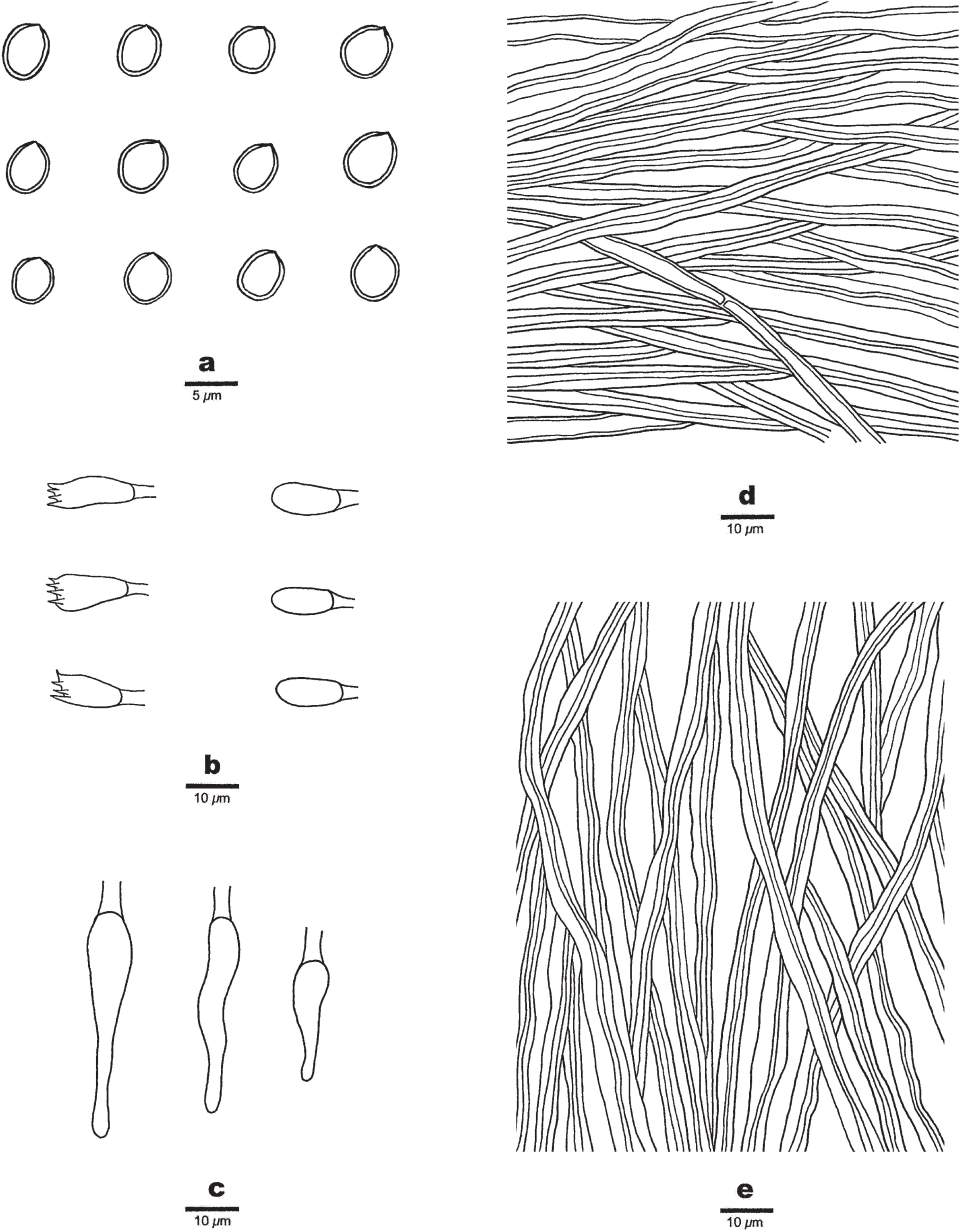
Microscopic structures of *Fulvifomesbolivianus* (Holotype, JV 2311/6-J). **a.** Basidiospores; **b.** Basidia and basidioles; **c.** Cystidioles; **d.** Hyphae from context; **e.** Hyphae from tube trama.

#### Etymology.

*Bolivianus* (Lat.): refers to the type of species being found in Bolivia.

#### Description.

Basidiomata perennial, pileate, solitary, without distinctive odor or taste, and woody hard when fresh, light in weight when dry. Pilei ungulate, projecting up to 11 cm and 5 cm wide and 2.5 cm thick at base. Pileal surface olivaceous when dry, encrusted, rough, concentrically sulcate, irregularly cracked; pileal margin brown, obtuse. Pore surface umber, with a reddish tint, unglancing; sterile margin distinct, fulvous, up to 3 mm wide; pores circular, 7–8 per mm; dissepiments thick, entire. Context umber, woody hard, zonate, up to 5 mm thick. Tubes fulvous, woody hard, up to 1 mm long, tube layers indistinctly stratified.

#### Hyphal structure.

Hyphal system dimitic in trama and context; generative hyphae simple septate; tissue becoming blackish brown in KOH.

#### Context.

Generative hyphae hyaline to brown, thick-walled, unbranched, simple septate, 2–2.5 µm in diameter; skeletal hyphae dominant, golden yellow to brown, thick-walled, unbranched, aseptate, more or less straight, regularly arranged, 4–6.5 µm in diameter.

#### Tubes.

Generative hyphae hyaline to brown, thick-walled, unbranched, simple septate, 2–3 µm in diameter; skeletal hyphae frequent, golden yellow to brown, thick-walled with a narrow lumen, unbranched, aseptate, more or less straight, subparallel along tubes, 2.5–4 µm in diameter. Setae or setal hyphae absent; cystidioles thin-walled, hyaline, varies in shape, fusoid to ventricose with elongated apical portion, 8.5–18 × 3.5–5.5 μm; basidia clavate to barrel-shaped, with four sterigmata and a simple basal septum, 15–18 × 6–7 µm; basidioles in shape similar to basidia, slightly smaller than basidia.

Basidiospores subglobose, yellowish brown, thick-walled, smooth, IKI–, CB–, (4.4–)4.7–5.4(–5.7) × (3.5–)4–5(–5.3) µm, L = 5.04 µm, W = 4.50 µm, Q = 1.12 (n = 30/1).

#### Type of rot.

White rot.

#### Additional specimen examined

**(paratype).** Bolivia. • St. Cruz Dept., Refugio los Volcanes, altitude 1100 M, dead angiosperm tree, 1.XI.2024, leg J. Vlasák Jr., JV 2411/15J.

### ﻿ Fulvifomes fragilis

Taxon classificationFungiHymenochaetalesHymenochaetaceae

Jian Chen, Yuan Yuan, K.Y. Luo, Y.C. Dai & Vlasák sp. nov.

F35A0F66-9341-55A1-8D8E-AD2C62E89330

858481

Figs 4 , 5

#### Diagnosis.

*Fulvifomesfragilis* is related to *F.luteoumbrinus* (Romell) Y.C. Dai & Vlasák, but *F.luteoumbrinus* differs from *F.fragilis* by a monomitic hyphal system and larger basidiospores (5.0–6.0 × 4.0–5.5 µm vs. 3.9–5.1 × 3–3.7 µm).

**Figure 4.**
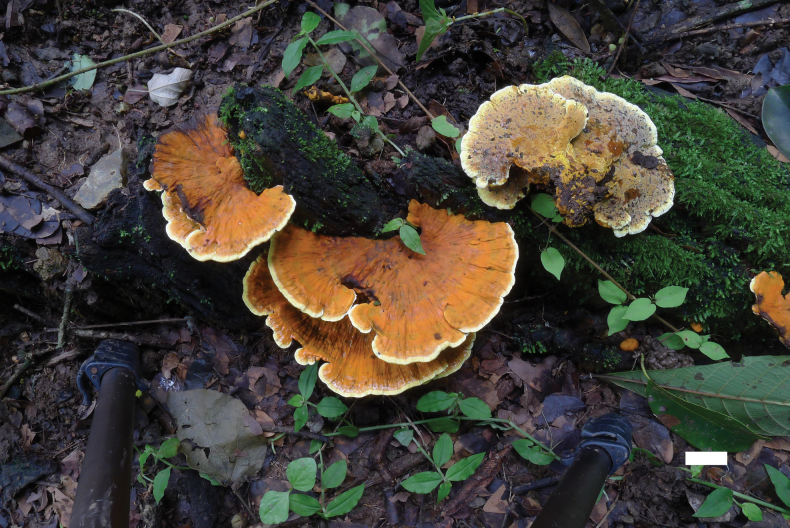
Basidiomata of *Fulvifomesfragilis* (Holotype, JV 2402/50J-1). Scale bar: 1 cm.

#### Holotype.

Ecuador. • Macará, Reserva Jorupe, dry tropical forest, altitude 650 m, on roots of rotten angiosperm tree, 21.II.2024, leg J. Vlasák Jr., JV 2402/50-J-1 (BJFC 053708-1).

**Figure 5.**
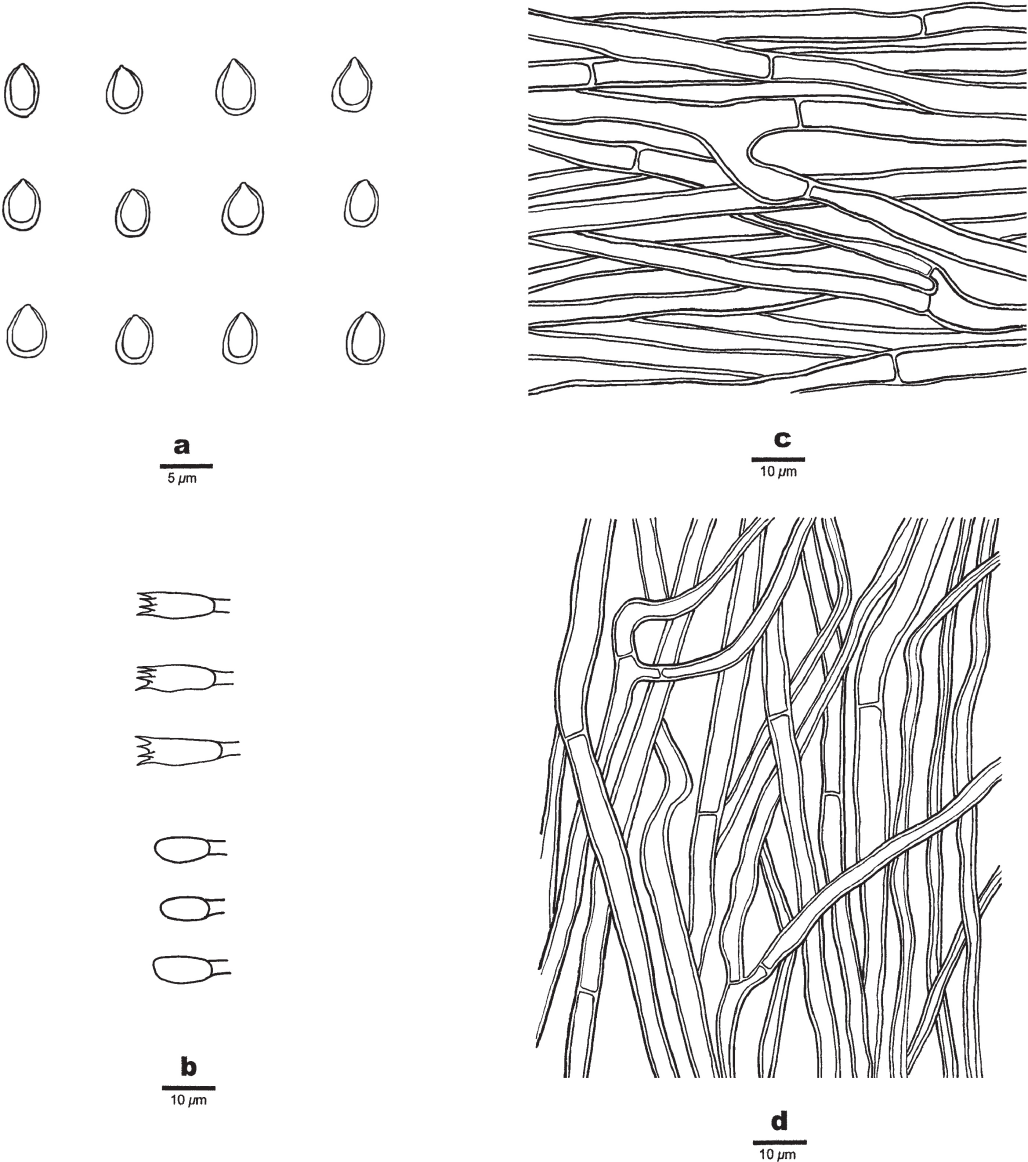
Microscopic structures of *Fulvifomesfragilis* (holotype, JV 2402/50-J-1). **a.** Basidiospores; **b.** Basidia and basidioles; **c.** Hyphae from context; **d.** Hyphae from tube trama.

#### Etymology.

*Fragilis* (Lat.): refers to the species having brittle basidiomata when dry.

#### Description.

Basidiomata annual, pileate, imbricate, without distinctive odor or taste, and woody hard to brickle when dry. Pilei sessile, projecting up to 7 cm and 11 cm wide and 8 mm thick at base. Pileal surface curry yellow to yellowish brown when dry, rough; pileal margin yellowish brown, narrow. Pore surface yellow, rugged; sterile margin distinct, yellow, up to 4 mm wide; pores circular to elliptical, 5–6 per mm; dissepiments thick, entire. Context lemon yellow, woody hard, up to 6 mm thick. Tubes concolorous with context, woody hard, up to 0.5 mm long.

#### Hyphal structure.

Hyphal system dimitic in trama and context; generative hyphae simple septate; tissue becoming blackish brown in KOH.

#### Context.

Generative hyphae pale yellow, thick-walled, occasionally branched, frequently simple septate, 3–4 µm in diameter; skeletal hyphae dominant, yellowish to golden yellow, thick-walled, unbranched, aseptate, more or less straight, regularly arranged, 3–4 µm in diameter.

#### Tubes.

Generative hyphae pale yellow, thick-walled, rarely branched, frequently simple septate, 2.5–4.5 µm in diameter; skeletal hyphae frequent, yellowish to golden yellow, thick-walled with a narrow to wide lumen, unbranched, aseptate, more or less straight, subparallel along tubes, 2.5–4.5 µm in diameter. Setae or setal hyphae absent; cystidioles absent; basidia clavate to barrel-shaped, with four sterigmata and a simple basal septum, 11–14 × 5–6 µm; basidioles in shape similar to basidia, slightly smaller than basidia.

Basidiospores broadly ellipsoid, yellowish brown, thick-walled, smooth, some collapsed, IKI–, CB–, 3.9–5.1 × (2.9–)3–3.7(–3.8) µm, L = 4.38 µm, W = 3.38 µm, Q = 1.30–1.32 (n = 60/2).

#### Type of rot.

White rot.

#### Additional specimen examined

**(paratype)**. Ecuador. • Macará, Reserva Jorupe, dry tropical forest, altitude 650 m, on roots of rotten angiosperm tree, 21.II.2024, leg J. Vlasák Jr., JV 2402/50-J-2 (BJFC 053708-2).

## ﻿Discussion

Most *Fulvifomes* species have relatively uniform basidiospores and lack setae, making morphological determination problematic (Dai 2010; Wu et al. 2022). DNA-based phylogenetics, however, provides a powerful tool that enables the discrimination of closely related species. Based on both phylogenetic analyses and morphology, we have described *Fulvifomesfragilis* and *Fulvifomesbolivianus* as new to science in this study. They fit well in *Fulvifomes*, with annual to perennial, pileate basidiocarps, broadly ellipsoid basidiospores, and the absence of setae or setal hyphae. Phylogenetically, they formed distinct lineages within the *Fulvifomes* clade based on ITS + nLSU datasets (Fig. 1).

Morphologically, *Fulvifomesbolivianus* is most similar to *F.krugiodendri* Y.C. Dai, X.H. Ji & Vlasák by sharing perennial and pileate basidiocarps, ungulate and cracked pilei, a dimitic hyphal system, and thick, entire dissepiments. Moreover, they have approximately the same size pores and basidiospores. *F.krugiodendri* differs from *F.bolivianus* by its dark gray pileal surface, grayish brown, glossy pore surface, and its distribution in Florida, USA (Ji et al. 2017). Phylogenetically, *F.bolivianus* is related to *F.squamosus* Salvador-Montoya & Drechsler-Santos, but *F.squamosus* is different from *F.bolivianus* by applanate to triquetrous pilei, a monomitic hyphal system in context, and larger pores (5–6 per mm vs. 7–8 per mm) (Salvador-Montoya et al. 2018).

*Fulvifomesfragilis* is phylogenetically related to *F.luteoumbrinus* (Romell) Y.C. Dai & Vlasák (= *Inocutisporrecta* (Murrill) Baltazar), a somewhat aberrant species within *Fulvifomes* for which, at one time, the genus *Aurificaria* (Romell) D.A. Reid was established due to its trametoid basidioma and very dark spores. Phylogenetic analysis revealed, however, a close relationship with *Fulvifomes* species, and it was accepted as *Fulvifomes* with a unique combination of features occurring in different species of *Fulvifomes* (Wu et al. 2022). *F.luteoumbrinus* differs from *F.fragilis* by a monomitic hyphal system and larger basidiospores (5–6 × 4–5.5 µm vs. 3.9–5.1 × 3–3.7 µm) (Baltazar et al. 2010; Sakayaroj et al. 2012). Morphologically, *Fulvifomesfragilis* resembles *F.indicus* (Massee) L.W. Zhou by sharing annual, pileate basidiocarps, approximately the same size pores, and the absence of setae or setal hyphae, but *F.indicus* has a monomitic hyphal system, larger basidiospores (5.4–6.5 × 4.7–5.5 μm vs. 3.9–5.1 × 3–3.7 µm), and a distribution in Southeast Asia and Australia (Zhou 2014). In addition, although *F.fragilis* shares a bright yellow color changing red with KOH and thin, flabelliform, often semistipitate fruitbodies with *Flaviporellussplitgerberi* (Mont.) Murrill (≡ *Inonotussplitgerberi* (Mont.) Ryvarden) (Wu et al. 2022), it forms a distinct clade within the *Fulvifomes* phylogeny.

In the phylogeny of Hymenochaetaceae, *Fulvifomes* is related to *Flaviporellus* and *Phylloporia*, and the three genera share the absence of setae and thick-colored basidiospores. However, *Flaviporellus* has basidiocarps becoming vivid deep red in KOH and collapsing basidiospores when mature, while *Phylloporia* grows almost always parasitically on living plants and has a duplex context. *Fulvifomes* usually grows on dead angiosperm trees or wood, has basidiocarps with homogeneous context that turn blackish brown in KOH, and its mature basidiospores are mostly not collapsed (Wu et al. 2022). In addition, most *Fulvifomes* species occur in tropical or subtropical areas (Wu et al. 2022); only *F.rimosus* (Berk.) Fiasson & Niemelä has been recorded in Europe (Ryvarden and Melo 2014). The two new species of *Fulvifomes* from South America discovered in this study also grow on dead angiosperm trees (*F.bolivianus*) or rotten wood (*F.fragilis*), and *F.fragilis* has collapsed basidiospores that become yellowish brown in KOH.

## Supplementary Material

XML Treatment for Fulvifomes bolivianus

XML Treatment for Fulvifomes fragilis

## ﻿Additional information

### Use of AI

No use of AI was reported.

### Author ORCIDs

Yuan Yuan https://orcid.org/0000-0001-6674-9848

Kai-Yue Luo https://orcid.org/0000-0001-6145-4864

Josef Vlasák https://orcid.org/0000-0002-5363-4459

### Data availability

All of the data that support the findings of this study are available in the main text.
